# Physiological responses of *Amaranthus cruentus* L. to drought stress under sufficient- and deficient-nitrogen conditions

**DOI:** 10.1371/journal.pone.0270849

**Published:** 2022-07-06

**Authors:** Inês Cechin, Laura Prado da Silva, Elisa Teófilo Ferreira, Sarah Corrêa Barrochelo, Fernanda Pereira de Souza Rosa de Melo, Anne Ligia Dokkedal, Luiz Leonardo Saldanha

**Affiliations:** Department of Biological Sciences, Faculty of Sciences, UNESP – São Paulo State University, Bauru, Brazil; United Arab Emirates University, UNITED ARAB EMIRATES

## Abstract

Water and nitrogen availability are two major environmental factors that can impair plant growth, and when combined, their effects on plant performance can be either intensified or reduced. The objective of this study was to analyze the influence of nitrogen availability on the responses of *Amaranthus cruentus*’s metabolism to water stress. The plants were cultivated in plastic pots filled with vermiculite, kept under greenhouse conditions, and were watered three times a week with 70% of a full strength nitrogen-free Long Ashton solution, containing 1.97 or 9.88 kg N ha^−1^ as ammonium nitrate. Photosynthetic parameters were evaluated *in planta*, and leaves were harvested for chemical analysis of photosynthetic pigments, proline, and phenolic contents. Higher nitrogen supply increased the shoot dry matter, photosynthetic pigments, photosynthesis, stomatal conductance, transpiration, total leaf nitrogen, proline, nitrate, and ammonium but reduced the concentration of flavonoids and total phenols. Six days of water stress did not affect dry matter, photosynthetic pigments, leaf nitrogen, ammonium, or specialized metabolites but increased the proline under high nitrogen and negatively affected stomatal conductance, transpiration, photosynthesis, relative water content, instantaneous water use efficiency, and leaf nitrate. The negative effect was more pronounced under high nitrogen supply. The results show that the addition of a high amount of nitrogen made the physiological processes of plants more sensitive to water stress, indicating that the plant response to water restriction depends on the interaction between the different environmental stressors to which the plants are subjected.

## Introduction

Plant growth is subject to the influence of several environmental stressors, which can lead to losses in agricultural productivity [1, 2]. Plants grow better in soils that contain adequate levels of nutrients for growth [3]. Nitrogen is an essential nutrient for plant development, and nitrogen deficiency reduces growth and photosynthesis because the large amount of total leaf nitrogen is located in the chloroplasts [4]. Mu and Chen [5] demonstrated that C_4_ plants invest more nitrogen into light-harvesting proteins, suggesting that these plants have a higher light energy convention and electron transport rate. A higher leaf nitrogen allocation is expected in most plants that are well-nourished with nitrogen and in a positive correlation with photosynthetic capacity in both C_3_ and C_4_ plants [6, 7]. Because of increased global warming, climate change, and the risk of extreme events such as prolonged droughts that are expected to increase in the coming years, rural areas are estimated to see major impacts on their water availability and supply [8], resulting in losses in agriculture. It is well known that water deficits are considered one major abiotic stress factor affecting plant productivity because of its negative effects on plant growth [9] and photosynthesis [10, 11]. Under mild water stress, the leaves close their stomata to save water and improve water-use efficiency. However, with the closure of the stomata, there is also a reduction in CO_2_ supply to the leaves, and as a result, there is a reduction in the capacity for CO_2_ assimilation.

Plants under water restriction can synthesize and accumulate several osmolytes in the cells to reduce their osmotic potential, thus enhancing their osmoprotection and regulation of the antioxidant defense system [12]. In a recent review about osmolyte synthesis and accumulation, it was shown that the osmoprotectant belongs to several classes of compounds, such as sugars, polyamines, and amino acids such as proline with all these osmolytes involved in reactive oxygen species (ROS) scavenging, osmotic adjustment, improving assimilation of CO_2_, and protecting cell membranes [13, and references therein]. Therefore, osmolytes have been associated with abiotic stress tolerance in plants. As suggested by Molla [14], the higher accumulation of proline might be helpful for better osmotic maintenance under water stress. Although proline is regarded as an osmoprotectant under several stress conditions, there is a recent theory that proline accumulation is linked with the detoxification of ROS [15]. In this case, the plants produce a large variety of specialized metabolites that appear to have no direct function in their growth or development but can function as a form of defense against abiotic stresses. Specialized metabolites, such as phenol and flavonoids, are accumulated under drought stress in some species [16–18], suggesting that they may be involved in the ROS-scavenging system and drought resistance.

The species *Amaranthus cruentus* is important as a nutritional food because both the leaves and seeds can be consumed. The leaves of the young *A. cruentus* plants can be used in salads and soups, and the grains are utilized in the production of breads, cakes, and cookies and can be added to soups [19]. In addition to the important nutritionally primary metabolites, amaranth plants also contain some specialized metabolite compounds that play an important role in the human diet [20]. Because of the highnutritional value of *A. cruentus*, the consumption of this plant has been recommended for its ability to contribute to different benefits, such as antioxidant activity, increased pro-vitamin A, and anticancerogenic compounds, all of which can help in a healthy diet [21]. Although *Amaranthus* has been described as drought resistant [22], water stress can affect its performance [23]. Data on the effect of drought and nitrogen availability on plant performance when applied alone have been well reported, but less information is available on their interactive effects. Because adequate nitrogen supply is necessary for better plant performance, we expect that *Amaranthus* could cope better with the effects of water stress. Because plant responses depend on the interaction between different environmental stresses, the aim of the current study was to analyze the influence of nitrogen availability on the responses of *A. cruentus* to water stress. We hypothesized that (1) both nitrogen and water stress would affect the physiological responses of amaranth plants, and (2) nitrogen supply would modify the effects of water stress on amaranth plants. Additionally, we evaluated the ability of plants to recover from water stress after 24 h of rehydration. The results can help in understanding the responses of amaranth plants to future climate change in relation to water availability.

## Materials and methods

### Plant material and growth conditions

BRS Alegria is a new cultivar of *A. cruentus* developed by the Center for Agricultural Research of Cerrados, which originated from the *A. cruentus* strain AM 5189 from the USA [24]. The seeds were purchased from the Brazilian Agricultural Research Company (Empresa Brasileira de Pesquisa Agropecuária—EMBRAPA), which is a Brazilian government company and the owner of the Center for Agricultural Research of Cerrados. The seeds of *A. cruentus* were sown in 4 L plastic pots filled with vermiculite and kept in a greenhouse under natural photoperiodic conditions and minimum and maximum average temperatures of 16 and 33°C, respectively. The plants were watered with 70% of full-strength, nitrogen-free Long Ashton solution [25], containing different doses of nitrogen as ammonium nitrate. The nitrogen doses used were as follows: 1.97 and 14.82 kg N ha^−1^, which correspond to 20% and 100% of full-strength Long Ashton nutrient solution, respectively. The plants were supplied with 300 mL of nutrient solution per pot three times a week and with water on the other days. The plants were stressed by suspension of watering after 42 days of growth. After six days of stress imposition, half of the stressed plants of both low and high nitrogen supply were rehydrated for 24 h to determine their ability to recover from water stress.

### Gas exchange measurements

A portable infrared gas analyzer (LCpro, ADC, Hoddesdon, UK) was used for photosynthesis measurement (*A*), stomatal conductance (*g*_*s*_), transpiration (*E*), and intercellular CO_2_ concentration (*C*_*i*_) on the youngest fully expanded leaf after six days of water stress imposition and after 24 h of rehydration. Measurements were taken between 8 and 10 a.m. inside the greenhouse under under ambient air temperature, carbon dioxide partial pressure and water vapor pressure. Photosynthetic active radiation (PAR) of 1000 *μ*mol m^−2^ s^−1^ was supplied by a light unit, which was mounted on top of the leaf chamber. The leaf was kept under this PAR until a steady-state rate was achieved. Instantaneous water use efficiency (WUE) was obtained as the ratio of photosynthetic carbon assimilation to water lost by transpiration (*A*/*E*).

### Relative water content determination

The leaf relative water content (RWC) was determined in four leaf disks of known area per plant after six days of water stress and after 24 h of rehydration according to the following equation:

$$\begin{array}{c} \text{RWC}\left(\%\right)=\frac{\left(\text{FW}-\text{DW}\right)}{\left(\text{TW}-\text{DW}\right)}\times 100\;, \end{array}$$ (1)

where FW is the fresh weight obtained immediately after removing the leaf discs; TW is the turgid weight determined after rehydration of the discs for 3 h; and DW is the dry weight obtained after drying the discs in an oven at 60°C for 48 hours.

### Photosynthetic pigments and leaf nutrients analysis

The photosynthetic pigments were measured on three leaf discs of known area from the same leaf used for gas exchange measurements. Pigments were extracted in 80% aqueous acetone, and the content was calculated according to the equations proposed by Lichtenthaler [26]. The proline content was determined according to the method described by Bates *et al*. [27] and modified by Torello and Rice [28] in oven dried and finely powdered leaves; the concentration was expressed on a leaf dry weight basis by using proline as the standard. The determination of nitrate, ammonium, and total nitrogen was made in the leaves located in the middle region of the plant. The leaves were oven dried at 60°C until constant weight was obtained. The leaves were then finely ground with a mill and sent for analyses at the Soil Laboratory of School of Agricultural Sciences, Botucatu, São Paulo/Brazil. Foliar nitrogen, nitrate, and ammonium analyses were performed by the semi-micro-Kjeldahl method, after sulfurous digestion of dried and finely ground leaves, here according to Malavolta *et al*. [29].

### Leaf phenolic content analysis

#### Extract preparation

Leaf samples from 24 specimens of *A. cruentus* grown in different cultivation conditions were collected in triplicate and hot air dried at 60°C. The dried leaves were powdered using a knife mill, and an aliquot of 50 mg was extracted using MeOH:H_2_O 85:15 (v/v) via ultrasound for 30 minutes at room temperature. The supernatant was collected and centrifuged at 3000 rpm. The obtained solution was subjected to solid phase extraction in the reverse phase (SPE-C_18_); the cartridges were filtrated by using a PTFE membrane (0.45 *μ*m). The samples were analyzed randomly at a final concentration of 3.5 mg ml^−1^.

#### HPLC-PAD analysis

The extracts were analyzed in a high-performance liquid chromatograph (PU-2089S Plus, Jasco®) coupled with a photodiode array detector (MD-2015 Plus Jasco®) and automatic injector (MD AS-2055 Jasco®). Chromatographic separations were performed on a Phenomenex® Luna C_18_ column (250 x 4.6 mm d.i., 5 *μ*m) at 40°C. The mobile phase system consisted of a MeOH and ultrapure water, acidified with 0.1% Formic Acid (Synth®). The samples were analyzed using a linear gradient, as follows: 5–65% of MeOH in ultrapure water for 65 minutes. The flow rate was 1 ml min^−1^.

#### Quantitative determination of constituents

The determination of the content of phenolic compounds was performed using the external standard method. Quantification of the constituents was performed using a regression curve, with each standard injected in triplicate. Measurements were performed at 280 for phenolic acids and 360 nm for flavonoids. The total phenol contents were obtained from the sum of the quantified values for phenolic acids and flavonoids, here in their respective extracts. Jasco ChromPass software (Version 1.8.1.6) was used to process the chromatograms.

### Dry matter determination

At the end of water stress treatment (49 days after sowing), the plants of each treatment type were selected randomly for shoot dry matter determination. The amaranth plants were divided into stem and leaves before being oven dried at 60°C for at least 48 h.

### Statistical analysis

Two-way ANOVAs (2x2) were used to assess the effects of nitrogen supply, drought, and their interactions in each variable, here by using SPSS/PC for Windows 9.0. When there was significant interaction (main effects and/or their interaction), comparisons among the treatments were made using Tukey’s test at 5%.

## Results and discussion

Water and nitrogen are the two main limiting factors for crop growth and productivity. As predicted, when the climate changes, there will be changes in water availability. The sensitivity of plants to water stress may be altered by factors such as nitrogen availability. Data on the interactive effect of nitrogen and water availability on the performance of amaranth plants are limited. Although the species of *Amaranthus* can survive in poor soil, in the current study, high nitrogen supply increased the aboveground dry matter by about 168% when compared with low nitrogen (Table 1). Six days of water stress were not enough to reduce dry matter of the aboveground matter, and no interaction between nitrogen and water stress was observed (Table 1).

**Table 1:** Shoot dry matter (g plant^−1^), photosynthetic pigment content (g m^−2^), relative water content (RWC; %), the two-way analysis of variance and the coefficient of variance of amaranth plants grown under low nitrogen and sufficient water supply (LN+W), low nitrogen and low water supply (LN-W), high nitrogen and sufficient water supply (HN+W), and high nitrogen and low water supply (HN-W) after six days of water stress and 24 h of rehydration. the values are the mean±SE of 10, 6, and 5 plants for shoot dry matter, photosynthetic pigments, and RWC, respectively.

Variables	End of stress				After 24 h of rehydration			
	LN+W	LN-W	HN+W	HN-W	LN+W	LN-W	HN+W	NH-W
Shoot DM	4.56±0.21	4.46±0.13	12.22±0.36	11.86±0.34	-	-	-	-
Chl *a*	0.108±0.004	0.103±0.004	0.326±0.012	0.293±0.014	-	-	-	-
Chl *b*	0.038±0.001	0.037±0.001	0.106±0.004	0.094±0.004	-	-	-	-
Chl *a*/*b*	2.858±0.024	2.795±0.017	3.076±0.046	3.105±0.051	-	-	-	-
Carotenoids	0.043±0.001	0.0445±0.002	0.108±0.004	0.100±0.004	-	-	-	-
RWC	91.0±2.3aA	91.4±1.5aA	96.7±0.5aA	84.2±4.2bA	92.7±0.6	94.0±0.2	92.8±0.5	91.6±1.1
Two-way ANOVA								
Source of variation	N	W	N*x*W		N	W	N*x*W	
Shoot DM	***	NS	NS		-	-	-	
Chl *a*	***	NS	NS		-	-	-	
Chl *b*	***	NS	NS		-	-	-	
Chl *a*/*b*	***	NS	NS		-	-	-	
Carotenoids	***	NS	NS		-	-	-	
RWC	NS	*	*		NS	NS	NS	
Coefficient of variation (%)								
Shoot DM	14.7	9.6	9.2	8.9	-	-	-	-
Chl *a*	9.0	9.3	9.2	11.8	-	-	-	-
Chl *b*	7.1	8.7	9.8	10.6	-	-	-	-
Chl *a*/*b*	2.1	1.5	3.7	4.0	-	-	-	-
Carotenoids	8.2	9.9	9.2	9.2	-	-	-	-
RWC	5.7	3.6	1.3	1.2	1.5	0.5	11.1	2.6

Significance levels are *, P<0.05; **, P<0.01; ***, P<0.001; NS, not significant,—not determined. The means followed by the same small letters (for W at a given N level) and capital letters (for N at a given W supply) are not significantly different at P = 0.05.

Nitrogen is a component of chlorophyll molecules. Therefore, an increase in the concentrations of these pigments is expected when the plants are supplied with more nitrogen. Chlorophyll *a* and chlorophyll *b* concentrations were increased by about 200% and about 189%, respectively (Table 1) when compared with low nitrogen. Also, the ratio *a*/*b* was increased by about 8% under high nitrogen as a result of a higher effect on chlorophyll *a* (Table 1); this suggests a higher Photosystem II activity. Carotenoids are accessory pigments for photosynthesis and play an essential role in the photoprotection of the photosynthetic apparatus. A higher nitrogen supply increased the concentration of carotenoids about 115% compared with low nitrogen. No effects of water stress and no interactions were found in these variables (Table 1). These data are opposite to those found in maize grown under nitrogen and water stress combination [30]. As shown by Caser *et al*. [31], the different levels of dehydrations can induce different responses in terms of chlorophyll and carotenoids content. Noteworthy here is that under prolonged drought, it was shown that the content of chlorophyll was not altered; however, the carotenoids were increased, demonstrating a protective effect of carotenoids on chlorophyll [32].

**Fig 1:**
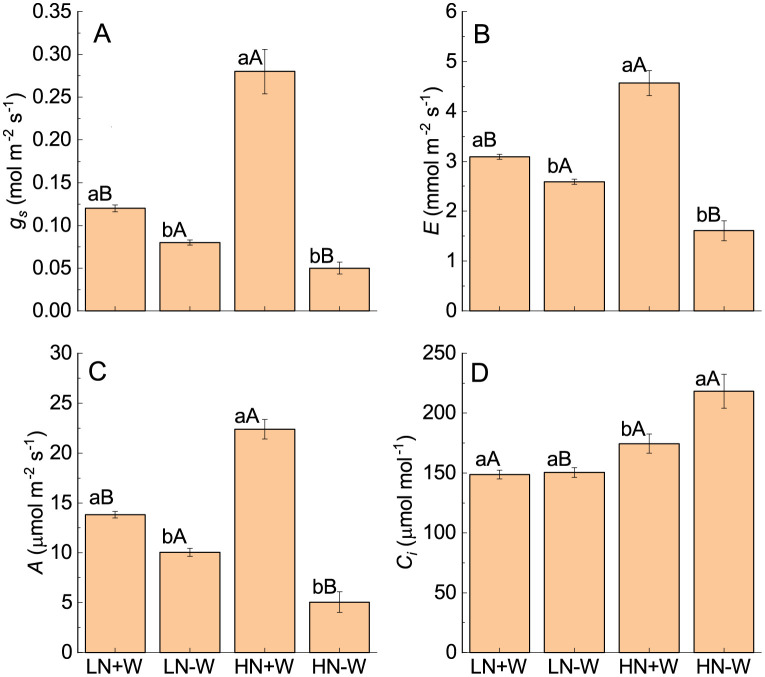
Stomatal conductance (*g*_*s*_, A), transpiration (*E*, B), photosynthesis (*A*, C) and intercellular CO_2_ concentration (*C*_*i*_, D) of amaranth plants grown under low nitrogen and sufficient water supply (LN+W), low nitrogen and low water supply (LN-W), high nitrogen and sufficient water supply (HN+W), and high nitrogen and low water supply (HN-W) after six days of water stress. The values are the mean±SE of seven plants. The means followed by the same small letters (for W at a given N level) and capital letters (for N at a given W supply) are not significantly different at *P* = 0.05.

The relative water content, which is an indication of the hydration status of the cells, was only affected by drought and its interaction (Table 1). Therefore, the relative water content was only reduced under high nitrogen, whereas lower nitrogen-grown plants maintained nearly the same relative water content as the control. This difference in relative water content between the low and high nitrogen supply under water restriction arises as a result of increased leaf biomass under high amounts of nitrogen. The greater leaf biomass under high nitrogen represents a greater area for transpiration and, as a result, faster consumption of water available in the substrate, in addition to stimulation of the rate of transpiration and stomatal conductance (Fig 1A and 1B). After 24 hours of rehydration, the relative water content of high nitrogen-grown plants recovered to the same level as the nonstressed plants (Table 1).

The response of stomata to nitrogen supply has been widely studied, but the available results are contradictory. In the present study, high nitrogen increased the stomatal conductance by about 133% of the plants grown under well water conditions (Fig 1A; Table 2) compared with low nitrogen. As observed by Matimati *et al*. [33], high nitrogen supply to roots enhances stomatal opening, provided that the plants are well watered. The restriction of the water supply reduced stomatal conductance under both nitrogen supply groups but because of the presence of an interaction between the two factors, there was a greater decrease in stomatal conductance under high nitrogen (78%) compared with low nitrogen (27%). In general, changes in stomatal conductance are reflected in changes in the measured rate of transpiration. Although nitrogen as the main factor did not significantly change the rate of transpiration, its interaction with water stress resulted in a higher negative (65%) effect of drought on the rate of transpiration (Fig 1B; Table 2), which is similar to the results found by Song *et al*. [34]. Studies have shown that high nitrogen supply resulted in a higher density of stomata [35], which may have also contributed to an increase in the rate of transpiration in the present study. The higher rate of transpiration per m^2^ and the higher leaf biomass produced under high nitrogen resulted in greater water loss through transpiration, causing a rapid exhaustion of available water in the substrate and a lower value of leaf relative water content. A small decline in stomatal conductance and rate of transpiration under mild water stress, here as observed under the low nitrogen condition, may have protective effects against drought by saving water because the relative water content under this condition was not altered after six days of water restriction (Table 1).

**Table 2:** The coefficient of variation and two-way ANOVA on several characteristics of amaranth plants grown under low nitrogen and sufficient water supply (LN+W), low nitrogen and low water supply (LN-W), high nitrogen and sufficient water supply (HN+W), and high nitrogen and low water supply (HN-W) after six days of water stress and 24 h of rehydration. Significance levels are as in Table 1.

Variables	End of stress				After 24 h of rehydration			
	LN+W	LN-W	HN+W	HN-W	LN+W	LN-W	HN+W	NH-W
Coefficient of variation (%)								
*g*_*s*_	8.3	8.5	24.5	40.2	11.3	4.3	13.9	10.3
*E*	6.5	5.6	14.5	33.1	8.2	3.4	22.6	9.4
*A*	6.3	10.6	11.5	54.0	13.0	3.8	14.8	12.4
*C*_*i*_	6.2	7.3	12.0	17.2	9.3	5.9	22.7	20.9
WUE	3.0	5.6	14.5	25.5	9.9	2.8	8.5	5.4
Proline	2.8	7.6	10.0	14.8	3.7	1.1	13.5	18.2
Leaf N	12.7	5.9	6.7	4.2	10.2	6.2	8.4	3.5
Ammonium	13.3	20.4	10.8	10.9	10.8	12.4	7.9	7.5
Nitrate	6.2	6.18	13.9	17.8	10.2	6.2	16.7	8.5
Two-way ANOVA								
Source of variation	N	W	N*x*W		N	W	N*x*W	
*g*_*s*_	**	***	***		***	NS	NS	
*E*	NS	***	***		***	**	*	
*A*	*	***	***		***	*	NS	
*C*_*i*_	***	*	*		NS	**	NS	
WUE	NS	***	**		***	NS	*	
Proline	***	*	*		***	NS	NS	
Leaf N	***	NS	NS		***	NS	NS	
Ammonium	***	NS	NS		***	NS	NS	
Nitrate	***	**	**		***	NS	NS	

Higher nitrogen supply had a significant stimulated effect of 163% on photosynthesis under well water supply, but the water stress drastically reduced it to values lower than in plants grown under a low nitrogen condition, here independently of water supply (Fig 1C). These results are in agreement with those obtained by Song *et al*. [34] in *Populus* species. The higher drought effect under a high nitrogen condition found in the current study indicates that *A. cruentus* would be more sensitive to water restriction under this condition than under low nitrogen supply, hence in line with the results found by Zhong *et al*. [36]. Evidence that appropriate water and fertilizer coupling regimes may improve the photosynthetic efficiency of plants and promote growth have been shown [37]. Therefore, choosing the appropriate nitrogen concentration may result in the enhanced drought resistance of plants [38]. However, the response to nitrogen and water supply depend on plant species, as found in *Phoebe zhennan*, where nitrogen fertilization plays a crucial role in alleviating water stress damage [39].

**Fig 2:**
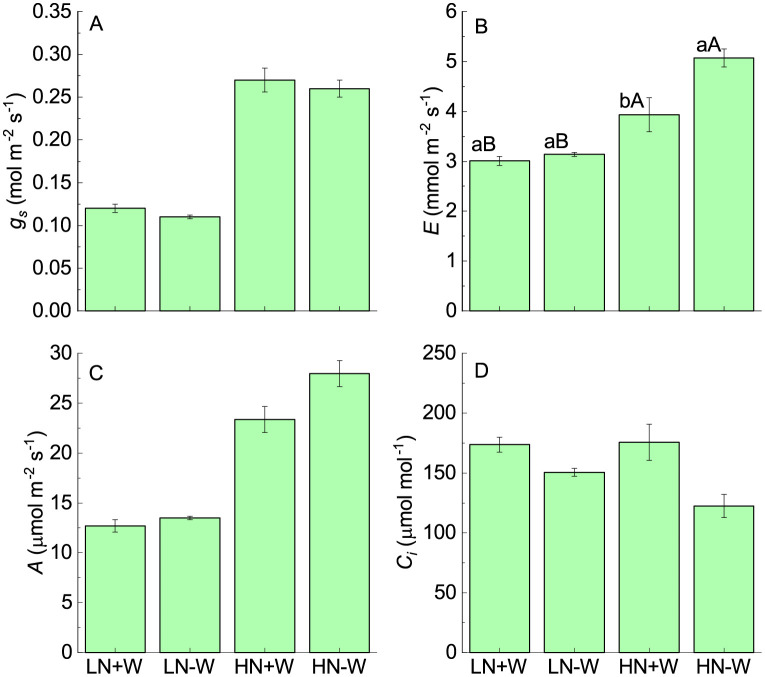
Relationship between photosynthesis (*A*) and stomatal conductance to the water vapor of amaranth plants grown under low nitrogen and sufficient water supply (LN+W), low nitrogen and low water supply (LN-W), high nitrogen and sufficient water supply (HN+W), and high nitrogen and low water supply (HN-W) after six days of water stress. The values are the mean±SE of seven plants. The regression equations are red square *y* = 6.2 + 62.3*x*, R^2^ = 0.75; black circle *y* = −1.3 + 139.7*x*, *R*^2^ = 0.90; blue up-pointing triangle *y* = 5.9 + 63.7*x*, *R*^2^ = 0.97; pink down-pointing triangle *y* = −1.6 + 137.2*x*, *R*^2^ = 0.98.

Photosynthesis had a positive linear relationship with stomatal conductance, hence representing the contribution of stomatal conductance on photosynthetic CO_2_ assimilation (Fig 2). The slope of the relationship between the two variables was much steeper under both nitrogen supply and drought compared with well water availability. Stomatal closure under water stress implies there is less CO_2_ available in the substomatal cavity for photosynthesis. If the stomatal conductance was exclusively limiting photosynthesis, the intercellular CO_2_ concentration would be expected to decrease under water limitation as a result of CO_2_ removal from the intercellular spaces and a reduction in the resupply because stomata are closed. Although the reduction in stomatal conductance could undoubtedly play a part in limiting CO_2_ fixation under stress, it is not the primary cause of the decline in photosynthetic activity under high nitrogen and probably under low nitrogen. On the contrary, under conditions of limited water, the intercellular CO_2_ concentration was increased by 127% under high nitrogen conditions and remained unchanged under low nitrogen conditions (Fig 1D), which is evidenced by the inhibition of photosynthesis not being attributed only to stomata closure but to other constraints on the CO_2_ photosynthetic assimilation capacity. The amount of intercellular CO_2_ concentration depends on the stomatal conductance, on the mesophyll conductance from substomatal cavities into the chloroplasts, and by metabolic processes. Although some authors have shown that water stress decreased the concentration of chlorophyll [34, 40, 41], which could be partially responsible for reducing photosynthesis, this is not the case in the present study. The inhibition of photosynthesis can occur because of a reduction in mesophyll conductance pathway from the substomatal cavities to the sites of carboxylation, hence resulting in lower chloroplastic CO_2_ partial pressures [42], in addition to a reduction in the photochemical efficiency of PSII, as found in maize, which is another C_4_ plant [30]. Reduced photosynthesis was also shown to be associated with a reduction in the activity of Rubisco and of the enzymes involved in the C_4_ pathway [43, 44] and with a lower relative water content in Rubisco activity [45]. As pointed out by Dabrowski *et al*. [10], the responses of photosynthesis to water stress are very complicated processes that include a reduction of the electron flow rate through PSII and inhibition of electron transfer from the reduced plastoquinone pool to the PSI reaction center, among others responses.

**Fig 3:**
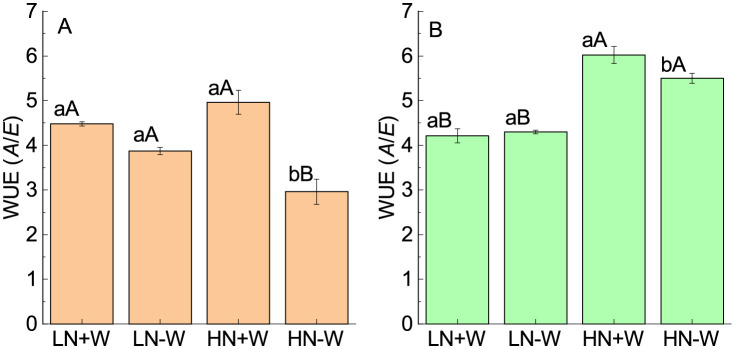
Stomatal conductance (*g*_*s*_, A), transpiration (*E*, B), photosynthesis (*A*, C) and intercellular CO_2_ concentration (*C*_*i*_, D) of amaranth plants grown under low nitrogen and sufficient water supply (LN+W), low nitrogen and low water supply (LN-W), high nitrogen and sufficient water supply (HN+W), and high nitrogen and low water supply (HN-W) after 24 h of rehydration. The values are the mean±SE of seven plants. The means followed by the same small letters (for W at a given N level) and capital letters (for N at a given W supply) are not significantly different at *P* = 0.05.

**Fig 4:**
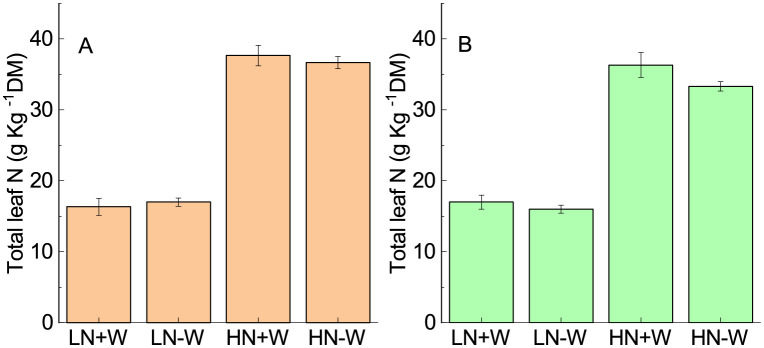
Instantaneous water use efficiency (WUE) of amaranth plants grown under low nitrogen and sufficient water supply (LN+W), low nitrogen and low water supply (LN-W), high nitrogen and sufficient water supply (HN+W), and high nitrogen and low water supply (HN-W) after six days of water stress (A) and 24 h of rehydration (B). The values are the mean±SE of seven plants. The means followed by the same small letters (for W at a given N level) and capital letters (for N at a given W supply) are not significantly different at *P* = 0.05.

After 24 h of rehydration, the stomatal conductance fully recovered under both low and high nitrogen supply, while the rate of transpiration under high nitrogen was increased about 29% when compared with nonstressed plants (Fig 3A and 3B; Table 2). The stimulation of the rate of transpiration under high nitrogen conditions could be explained as an attempt to reestablish the relative water content. The rate of photosynthesis fully recovered in low-nitrogen-grown plants after 24 h of rehydration, while in high-nitrogen-grow plants, photosynthesis showed a small but not significant increase when compared with its control (Fig 3C; Table 2). The intercellular CO_2_ concentration was reduced under low and high nitrogen supplies after 24 h of rehydration. The reduction that was found mainly under high nitrogen conditions can be explained by the stimulation of photosynthesis rapidly consuming the intercellular CO_2_ concentration available (Fig 3D; Table 2). The instantaneous water use efficiency was strongly reduced under water restriction and high nitrogen conditions as a result of a more negative effect on photosynthesis than on the rate of transpiration (Fig 4A; Table 2). After 24 h of rehydration, the instantaneous water use efficiency was not fully recovered because of the significant increase in the rate of transpiration compared with photosynthesis (Fig 4B). The fact that photosynthesis was fully recovered after rehydration indicates that damage to the photosynthetic apparatus did not occur under this level of water stress and the plants were able to repair their metabolic processes within a short time [10].

**Fig 5:**
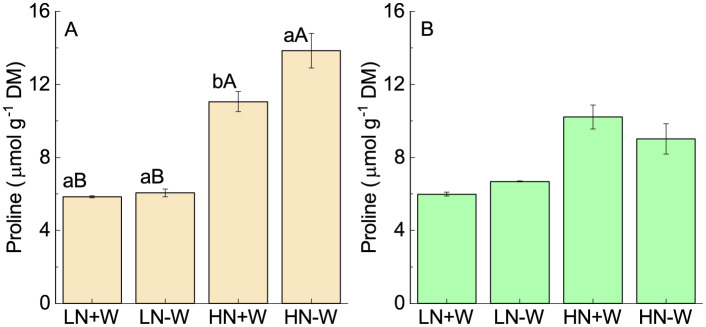
The total leaf nitrogen concentration of amaranth plants grown under low nitrogen and sufficient water supply (LN+W), low nitrogen and low water supply (LN-W), high nitrogen and sufficient water supply (HN+W), and high nitrogen and low water supply (HN-W) after six days of water stress (A) and 24 h of rehydration (B). The alues are the mean±SE of three plants.

In high-nitrogen-grown plants grown under water limiting conditions, the total leaf nitrogen content increased 232% (Fig 5A; Table 2), while stomatal conductance and photosynthesis increased about 237% and 62%, respectively compared with those plants grown under a low nitrogen supply (Fig 1A and 1C). Stomatal conductance has a positive relationship with leaf nitrogen [35]. Therefore, the stimulation of photosynthesis can be attributed to the increase in stomatal conductance, the high investment of nitrogen into the photosynthetic machinery, such as photosynthetic pigments, chloroplast development [46], and more nitrogen allocation into the photosynthetic enzymes Rubisco and PEPcase, as observed in amaranth plants by Tazoe et al. [47]. Drought stress was reported to significantly decrease leaf nitrogen [48, 49], but this finding is inconsistent with those observed in the current study. Because the restriction of water did not reduce the photosynthetic pigments or the leaf nitrogen content, the results suggest that a dysfunction in photosynthesis is not a result of lower leaf nitrogen or the presence of less photosynthetic pigments, but rather, it is because of biochemical and/or photochemical limitations, in addition to any stomatal conductance. It is interesting that under sufficient nitrogen conditions, the plants that present high levels of leaf nitrogen were not able to cope with the effect of water stress. It seems that here, the lower relative water content in the leaves may compromise the activity of the enzymes involved in photosynthesis, as pointed out by some authors [43–45]. The mechanistic basis for the lower rates of photosynthesis under high nitrogen and water stress has yet to be elucidated for amaranthus. After 24 h of rehydration, no change in leaf nitrogen content was observed when compared with the stressed conditions (Fig 5B).

**Fig 6:**
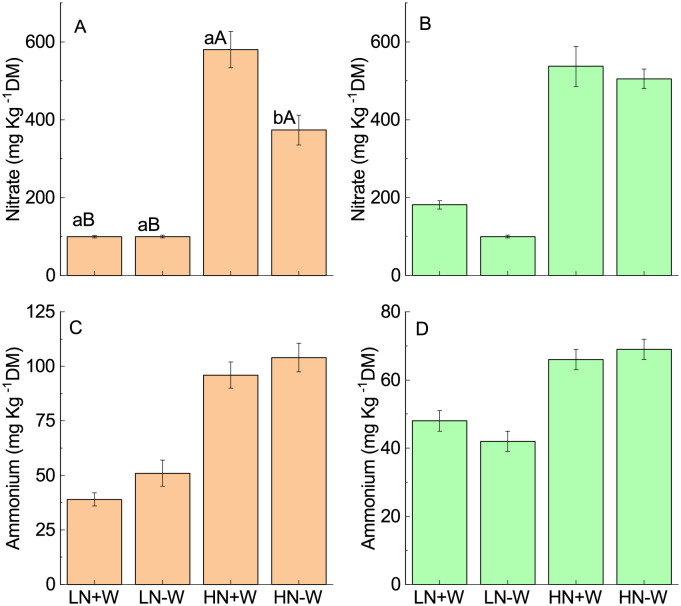
Proline concentration of amaranth plants grown under low nitrogen and sufficient water supply (LN+W), low nitrogen and low water supply (LN-W), high nitrogen and sufficient water supply (HN+W), and high nitrogen and low water supply (HN-W) after six days of water stress (A) and 24 h of rehydration (B). The values are the mean±SE of four plants. The means followed by the same small letters (for W at a given N level) and capital letters (for N at a given W supply) are not significantly different at *P* = 0.05.

Proline works as both an osmoprotectant and redox-buffering agent, possessing antioxidant properties under conditions of stress [50]. The accumulation of proline under drought stress was found in several plants [41], particularly in young leaves [51]. The accumulation in proline becomes greater as the stress period increases [17], suggesting that a change in osmotic potential is important to keep various physiological processes functioning. Besides this, proline was found to be the major nonenzymatic antioxidant metabolite under water stress conditions, resulting in stress tolerance because of its ROS‑scavenging ability [52]. The potential role of proline on drought tolerance was recently shown by Ahammed *et al*. [53], who used SlWRKY81-silenced tomato plants. Because proline is an amino acid that contains nitrogen, an increase in its synthesis was expected under high nitrogen supplies (Fig 6A; Table 2). At lower nitrogen levels, the difference between well-watered and stressed plants was smaller and not significant. However, under high nitrogen supply a significant increase in proline was observed. It is not surprising that in low nitrogen conditions, there was no significant increase in proline because the water stress in this condition was slow, with no significant changes in RWC. Therefore, it likely that an increase in proline might be helpful for better osmotic maintenance under water stress. The beginning of the accumulation and amount of proline accumulated under drought was found to depend on the *Amaranthus* species and genotype’s sensitivity, along with the degree of water stress [40, 54] and nitrogen level, as found in rice leaves [55]. After 24 h of rehydration, the level of proline was reestablished, meaning that only the effect of nitrogen remained (Fig 6B; Table 2).

**Fig 7:**
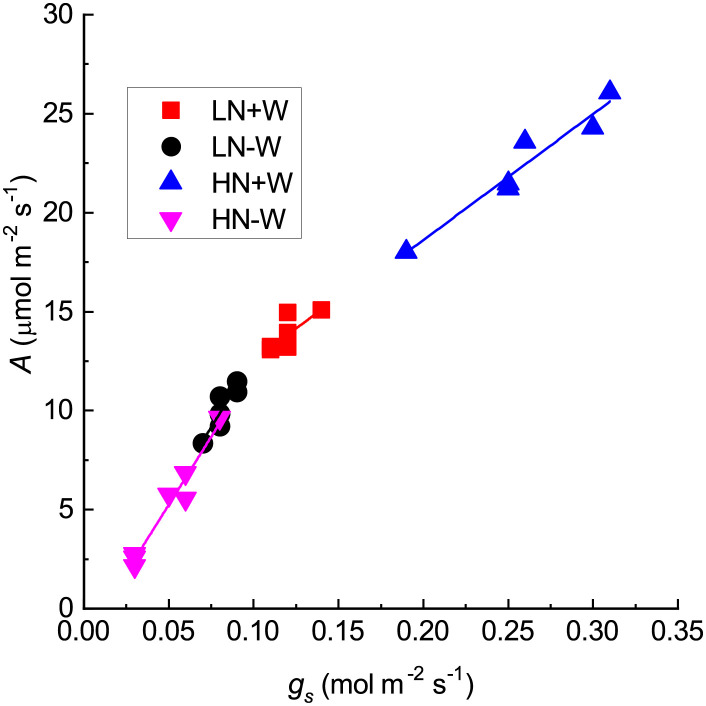
Leaf nitrate (A, B) and ammonium (B, C) concentrations of amaranth plants grown under low nitrogen and sufficient water supply (LN+W), low nitrogen and low water supply (LN-W), high nitrogen and sufficient water supply (HN+W), and high nitrogen and low water supply (HN-W) after six days of water stress (A, C) and 24 h of rehydration (B, D). The values are the mean±SE of three plants. The means followed by the same small letters (for W at a given N level) and capital letters (for N at a given W supply) are not significantly different at *P* = 0.05.

Nitrate and ammonium are the main nitrogen sources for plants, with nitrate being the preference of most species [56]. Plants supplied with a higher nitrogen supply show an increase in root hydraulic conductivity [57]. In the present study, large accumulations of leaf nitrate (582%) were found when the plants were supplied with higher nitrogen without water restriction (Fig 7A; Table 2). The increase may be due to an excess in absorption as a result of greater availability of nitrogen in the soil in relation to the transformation capacity into organic compounds. The leaves of *A. cruentus* presented an accumulation of nitrate of 579.5 mg kg^1^ of dry mass while under low nitrogen supply it was 99.5 mg kg^1^ of dry mass. Vacuoles are the major nitrate storage pool [58] which can be reutilized under conditions where the nitrogen supply is limited, such as a decrease of nitrogen uptake under water stress [55]. The total leaf nitrogen content was not affected by water stress at the two nitrogen levels tested (Fig 5A), but water stress increased proline and decreased leaf nitrate under high nitrogen conditions (Fig 5A; (Fig 6A), which is a response similar to that found for wheat [55]. After six days of water stress, high-nitrogen-grown plants attained a 36% lower concentration of nitrate than the corresponding leaves of nonstressed plants with no effect on low nitrogen (Fig 7A; Table 2). Although the plants received equal doses of nitrate and ammonium, the accumulation of ammonium in the leaves was 246%, while for nitrate, it was 582% (Fig 7C; Table 2). The increase in ammonium indicates saturation of the capacity of the enzymes involved in the assimilation of nitrogen into organic compounds. Although high levels of ammonium are considered toxic to plants, hence resulting in growth reduction [59], high levels of ammonium in the leaves of *A. cruentus* did not result in visible toxic effects after six days of water restriction.

Under water stress, the specific root water uptake and leaf hydraulic conductance are severely reduced [60, and references therein 61]. Drought can also alter nitrogen uptake and assimilation in plants because water is required for nitrogen absorption and utilization, suggesting that the plants can become simultaneously water and nitrogen limited [62]. Indeed, as observed by Wang *et al*. [63], higher stomata apertures in the drought-resistant cultivar of apple plants enhanced transpiration rate, which promoted more nitrogen uptake. A relatively higher xylem secretion rate and transpiration rate, which is the major pathway of water loss, were observed in moderate and high nitrogen supply conditions, but under water stress, they were significantly suppressed at all nitrogen levels [55], with no alteration in the total leaf nitrogen. As found by Zhong *et al*. [55], the greater reduction in *E* under the high nitrogen and water stress condition did not result in alterations of the total leaf nitrogen in amaranth, instead altering the concentrations of proline and nitrate. This may indicate that under a high nitrogen and water stress condition, there is a change in the allocation of nitrogen to proline and other nitrogen compounds. Water stress and nitrogen metabolism have interactive effects; consequently, nitrogen metabolism affects a series of physiological and biochemical changes that are of great significance for plant tolerance to drought [64]. The enzyme activity of nitrogen metabolism was higher under an adequate nitrogen supply when compared with low availability, but the activity of nitrate reductase was reduced by water stress under both nitrogen supply conditions [30]. Song *et al*. [34] observed that the nitrate content and nitrate reductase activity of maize leaves were significantly reduced under drought stress, while moderate nitrogen supply promoted the accumulation of nitrate and an increase in the nitrate reductase activity; they concluded that a moderate nitrogen supply increases plant resistance to drought stress, while high or low nitrogen concentrations increase the sensitivity of maize to drought stress.

Xiong *et al*. [48] found that there was a significant interactive effect between nitrogen and water stress on nitrate and ammonium content in the leaves of high-nitrogen-grown plants, which presented significantly lower and higher content under drought stress, respectively. The authors attributed the accumulation in ammonium in the leaves under drought and high nitrogen supply to an increment in nitrate reductase activity and a reduction of glutamine synthetase activity. This shows that an increased accumulation of ammonium under drought might be attributed to enhanced nitrate reduction. This is not the case in the current study because under high nitrogen and water restrictions, the lower nitrate content was not accompanied with an increase in ammonium in the leaves. It is likely that because of the lack of change in total leaf nitrogen, there was a deviation of nitrogen to proline synthesis. Slabbert [54] suggested that proline may act as a storage compound for carbon and nitrogen during water stress when the synthesis of starch and protein are inhibited. Additional research is needed to clarify this response in *A. cruentus* plants. There is abundant evidence that water deficits can alter the nitrogen metabolism, but the effect may vary accordingly to the species and intensity of stress. For example, in *Hippophae rhamnoides*, the two levels of drought stresses (50% and 30% field capacity for 12 weeks) decreased the absorption of ammonia and nitrate nitrogen, whereas only it inhibited the nitrate absorption of *H. thibetana* [65]. Also, as found by Huang *et al*. [66], the net influx of nitrate at the root surface was lower in response to drought stress, while the influx of ammonium tended to increase along the fine roots of PEG-treated *Malus prunifolia*. These findings explain the lower nitrate levels in the leaves of *A. cruentus* but not in the level of ammonium, which remained unchanged under drought stress. After 24 h of rehydration, the nitrate levels under high nitrogen were re-established (Fig 7B; Table 2). In the present study, neither water stress nor rehydration altered the level of ammonium in the leaves (Fig 7C and 7D; Table 2).

The available resources, such as nitrogen, carbon, and sulphur, will impact the production of specific classes of primary metabolites, which, in turn, synthesize specialized metabolites, hence indicating super-coordinated gene expression networks connecting the primary and specialized metabolism in plants [67]. The photosynthetic capacity is negatively correlated with the leaf phenolic concentration [68]; this has been suggested to represent the gradient between a maximum carbon gain and maximum protection [69], reflecting the trade-off between growth and protection demands, here depending on the growth strategy adopted by each species [68]. The phenolic compounds that are mainly found in the vacuole of several cell types in the leaves act as multifunctional specialized metabolites under abiotic stress [69], such as nonenzymatic antioxidants [70] and the repair of the membrane from lipid peroxidation [71]. Several authors have found that water-stressed plants show an increase in total phenols and flavonoids [31, 41], which suggests that the increased synthesis of these substances represents an important defense mechanism when it comes to drought tolerance [16]. As it has been pointed out, the biosynthesis of flavonoids occurs predominantly when the antioxidant enzymes are inactivated [72].

In the present study, the low- and high-nitrogen-grown plants of *A. cruentus* were stressed through stopping irrigation completely for six days. The representative chromatograms for each experimental group are shown in the (S1 Fig). Peaks in the chromatogram were identified as phenolic acids and flavonoids, here based on a comparison of absorption spectra in UV (S2 Fig) with the available standards. The areas of each of the detected peaks were obtained, the calculations extrapolated, and the quantification are expressed in *μ*g mg^−1^ of the dry extract. The average obtained for each experimental group and classes of substances are presented in (Table 3). The coefficient of variation, which is a measure of dispersion of the variables, were, in general, high (Table 3), which means that the higher the coefficient of variation, the greater the level of dispersion around the mean. The two-way ANOVA presented in Table 3 shows that the water stress did not significantly change the content of phenolic acids, total phenols, or flavonoids. The reason for this response might be because of the duration and intensity of the stress, as pointed out by Mahajan *et al*. [73]. On the contrary, high levels of nitrogen reduced the concentration of flavonoids and total phenols independently of water stress. The total phenol concentration of *Beta vulgaris* plants under N-starvation conditions was higher in the leaves and roots when compared with standard nitrogen supply [74]. This result is similar to what was found in the current study for amaranth, which belongs to the same family. The effect of increasing nitrogen on *Lactuca sativa* plants not only reduces the content of phenolic compounds, but also decreases every type of phenolic compound [75]. Because photosynthetic capacity is negatively correlated with the leaf phenolic concentration [68], this can partially explain the lower concentration of the total phenols and flavonoids under a high nitrogen supply found in the current study. After 24 h of rehydration, only nitrogen had a small significant effect on the content of phenolic acids and flavonoids (Table 3). The content of phenolic acids presented a small increase, while flavonoids remained lower after 24 h of rehydration when compared with those plants with a low nitrogen supply.

**Table 3:** Content of phenolic acids, flavonoids, and total phenols in *μ*g/mg of 85% MeOH leaf extract, the coefficient of variation, and ANOVA of amaranth plants grown under low nitrogen and sufficient water supply (LN+W), low nitrogen and low water supply (LN-W), high nitrogen and sufficient water supply (HN+W), and high nitrogen and low water supply (HN-W) after six days of water stress (A) and 24 h of rehydration. Significance levels are as in Table 1.

Variables	End of stress				After 24 h of rehydration			
	LN+W	LN-W	HN+W	HN-W	LN+W	LN-W	HN+W	NH-W
Fenolic acids	26.91±1.46	42.17±2.76	33.41±1.94	29.73±9.09	21.28±5.51	24.62±2.75	31.19±3.51	32.42±0.77
Flavonoids	53.01±1.95	44.96±2.92	32.91±1.13	26.02±6.94	36.08±8.90	38.27±2.26	24.99±2.48	20.99±7.47
Total phenols	80.01±0.55	87.13±4.59	66.32±0.81	55.75±14.56	57.37±8.55	62.89±5.01	56.18±5.75	53.41±7.84
Coefficient of variation (%)								
Fenolic acids	9.4	11.3	10.0	52.9	44.8	19.7	19.5	4.1
Flavonoids	6.4	11.2	5.9	46.0	42.7	10.2	17.2	61.2
Total phenols	1.2	9.0	2.1	45.0	25.8	13.8	17.0	25.4
Two-way ANOVA								
Source of variation	N	W	N*x*W		N	W	N*x*W	
Phenolic acids	NS	NS	NS		*	NS	NS	
Flavonoids	**	NS	NS		*	NS	NS	
Total phenols	*	NS	NS		NS	NS	NS	

## Conclusion

Exposure of *A. cruentus* plants to a high nitrogen supply resulted in better performance. Indeed, under this satisfactory condition, the plants did not need to invest in secondary defense metabolites such as phenolic compounds, which were reduced under this condition. A short period of water restriction had a negative impact on the relative water content, gas exchange characteristics, instantaneous water use efficiency, and on leaf nitrate levels, but the proline concentration was increased, and there was no increase in the phenolic compounds. The combination of a short water restriction with high nitrogen supply resulted in a more severe negative effect on amaranth gas exchange characteristics than with low nitrogen, thus resulting in plants that were more sensitive to water restriction. All together, the current study suggests that the responses of amaranth to water stress are likely to be influenced by nitrogen status. More studies are needed to elucidate the mechanisms involved in amaranth responses to the interaction between nitrogen and water availability.

## Supporting information

S1 Fig Analytical chromatography. (PDF)

S2 Fig UV-Vis spectra. (PDF)
