# Supplementary material for: Physiological responses of Amaranthus cruentus L. to drought stress under sufficient- and deficient-nitrogen conditions
Source: PLoS One. 2022 Jul 6;17(7):e0270849. doi: 10.1371/journal.pone.0270849 (PMC9258897; doi:10.1371/journal.pone.0270849)
Supplement: S1 Fig — (PDF) [file pone.0270849.s001.pdf]

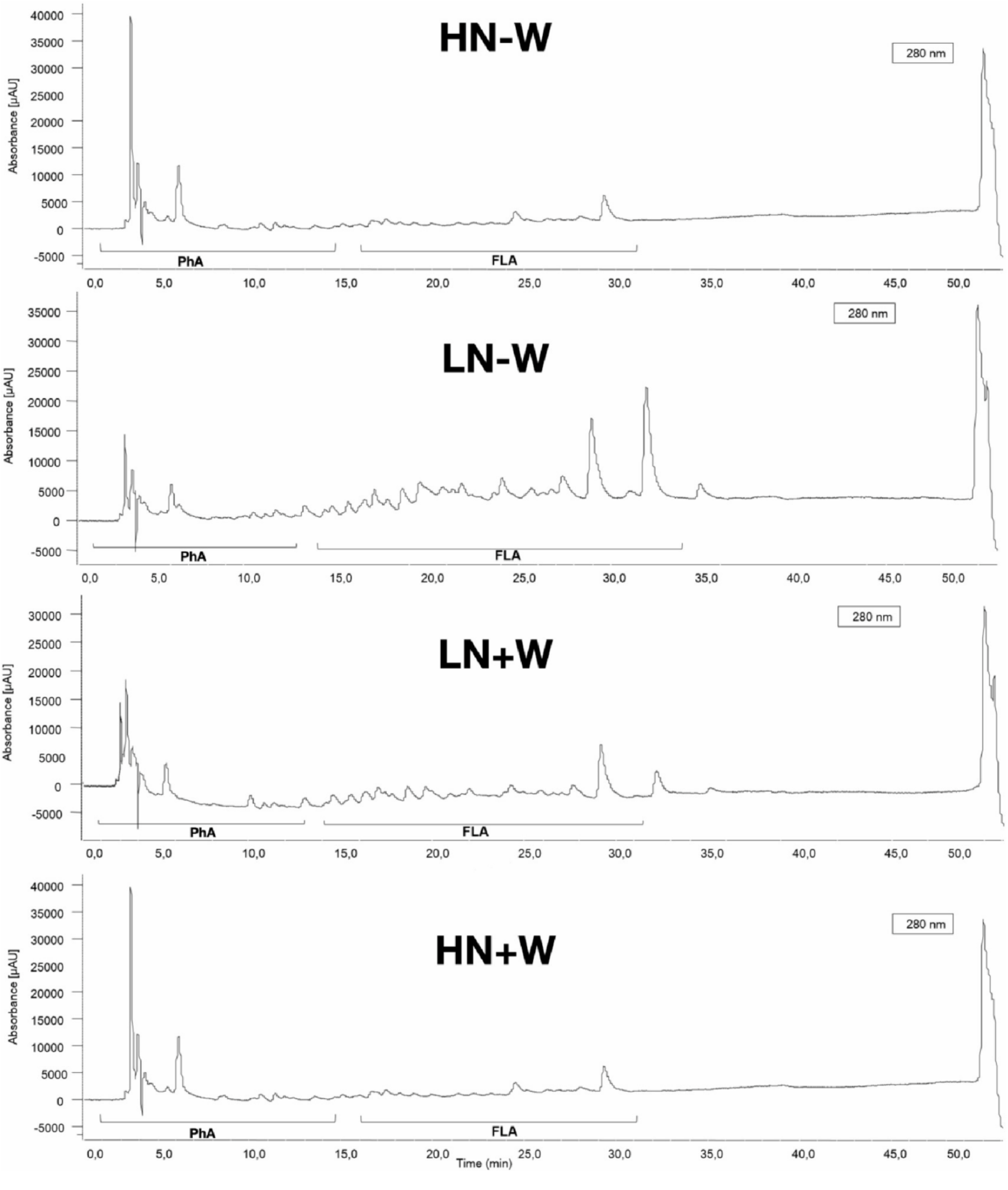

S1 Fig. Analytical chromatography. Representative analytical chromatograms obtained by HPLC-PAD from the 85% MeOH leaves extract of amaranth plants grown under different conditions of nitrogen and water supply. The peaks in the chromatogram were identified as phenolic acids (PhA) and as flavonoids (FLA) based on UV-vis absorption patterns by means of PAD analysis. Legend: high nitrogen and stressed (HN-W), low nitrogen and stressed (LN-W), low nitrogen and well hydrated (LN+W) and high nitrogen and well hydrated (HN+W) plants.
