# Supplementary material for: Physiological responses of Amaranthus cruentus L. to drought stress under sufficient- and deficient-nitrogen conditions
Source: PLoS One. 2022 Jul 6;17(7):e0270849. doi: 10.1371/journal.pone.0270849 (PMC9258897; doi:10.1371/journal.pone.0270849)
Supplement: S2 Fig — (PDF) [file pone.0270849.s002.pdf]

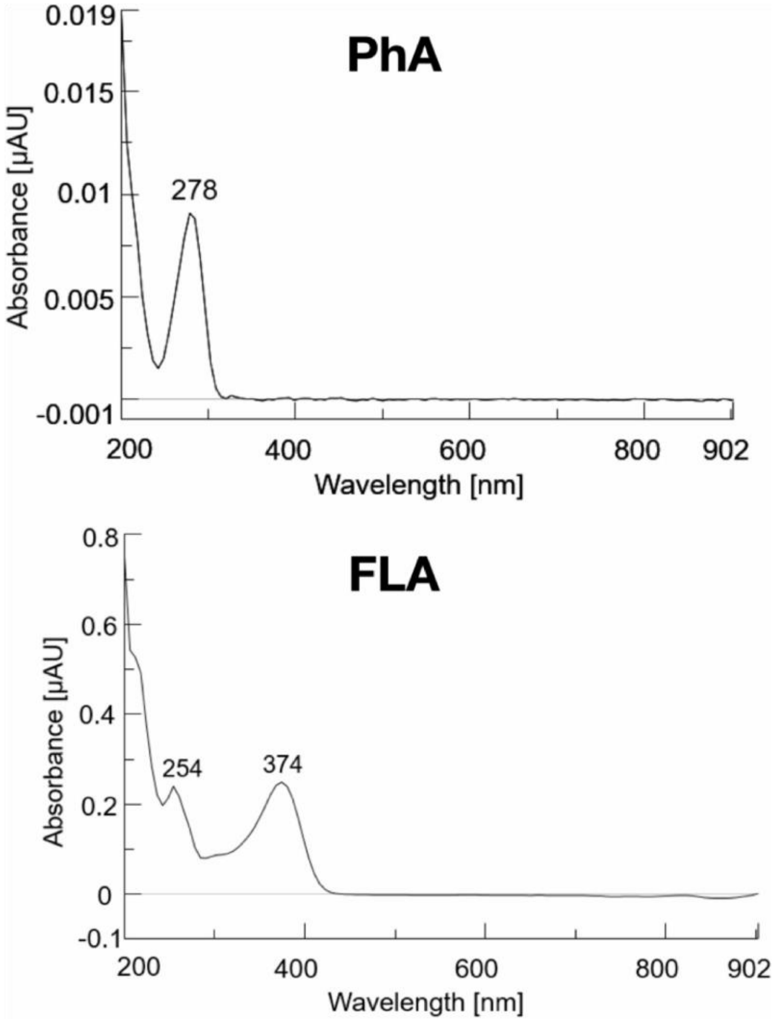

S2 Fig. UV-Vis spectra. Representative UV-vis spectra obtained by HPLC-PAD of peaks from the analytical chromatograms of amaranth plants 85% MeOH extracts assigned as phenolic acid (PhA) and as flavonoid (FLA) derivatives.
